# The perceived effects of faculty presence vs. absence on small-group learning and group dynamics: a quasi-experimental study

**DOI:** 10.1186/s12909-014-0258-1

**Published:** 2014-12-10

**Authors:** Miriam Hoffman, Joanne E Wilkinson, Jin Xu, John Wiecha

**Affiliations:** Department of Family Medicine, Boston University School of Medicine, One BMC Place, Boston, MA 02118 USA; Department of Community Health Sciences, Boston University School of Public Health, Boston, USA; Department of Medicine, Residency Training Programs, PO Box 208030, New Haven, CT 06520-8030 USA; Office of Medical Education, Boston University School of Medicine, Boston, USA

**Keywords:** Small-group learning, Group dynamics, Faculty presence, Medical education

## Abstract

**Background:**

Medical education increasingly relies on small-group learning. Small group learning provides more active learning, better retention, higher satisfaction, and facilitates development of problem-solving and team-working abilities. However, less is known about student experience and preference for different small groups teaching models. We evaluated group educational dynamics and group learning process in medical school clerkship small group case-based settings, with a faculty member present versus absent.

**Methods:**

Students completed surveys after cases when the faculty was present (“in”) or absent (“out”) for the bulk of the discussion. 228 paired surveys (114 pairs) were available for paired analysis, assessing group dynamics, group learning process, student preference, and participation through self-report and self-rating of group behaviors tied to learning and discussion quality.

**Results:**

Ratings of group dynamics and group learning process were significantly higher with the faculty absent vs. present (p range <0.001 to 0.015). Students also reported higher levels of participation when the faculty member was absent (p = 0.03). Students were more likely to express a preference for having the faculty member present after “in” case vs. “out” case discussions. (p < 0.001). There was no difference in reported success of the case discussion after “in” vs. “out” cases (p = 0.67).

**Conclusions:**

Student groups without faculty present reported better group dynamics, group learning processes, and participation with faculty absent. Students reported that they feel somewhat dependent on faculty, especially when the faculty is present, though there was no significant difference in students reporting that they obtained the most they could from the discussion of the case after both “in” and “out” cases.

## Background

Medical school educators are increasingly using small group, problem-based learning strategies [1], particularly in the pre-clinical years [2]. Small group learning appears to be associated with better retention of material [3,4], more active learning [5], and higher learner satisfaction [5]. Students who engage in small group learning also appear to have higher satisfaction with their education [6,7], better performance on both written exams [2] and objective standardized clinical exams (OSCEs) [7], especially when students arrive for small group work prepared, trained and knowledgeable. Small group learning may also help overcome cultural barriers and promote unified, collaborative learning among diverse student groups [8,9].

While the potential benefits of small group learning are many, it is unclear exactly why small group learning is successful [10]. The engagement of students in the group process and group dynamics appears important, though few students and faculty have a clear idea of what that means [11]. Researchers have had mixed results defining successful models of group learning by examining the respective roles of students and faculty in both student-led and faculty-led groups. Some researchers have demonstrated that an active role for the faculty leads to improved student satisfaction, increased USMLE scores, and increased perception by students of the faculty’s importance [12-15], particularly when the faculty member is well-versed in the subject matter and positively influences group functioning.

However, another study noted that faculty members were responsible for 63% of the interactions in faculty-led groups of veterinary students, who also followed a case-based curriculum. High numbers of faculty-led interactions can result in a teacher-dominated discussion [16]. Though the faculty member presumably has a much stronger command of the subject matter than students, teacher-dominated discussions may impede student leadership development and limit opportunities for interactive learning. In addition, students’ critical thinking may be impeded by the presence of an authority figure who is assumed to have information that they lack. These finding are inconsistent with the goal of engaging students in the group process. However, another study noted that student-led groups were more likely than faculty-led groups to use shortcuts [17], which may undermine the development of complex problem-solving skills. Additional studies reported either strong [18] or no relationships [16,19] between the skills of the faculty leader and the groups’ interaction and performance, suggesting that the effect of faculty leadership on small group learning may be highly variable.

Students’ perspectives on small-group learning are similarly varied. A majority of students ranked the role of the faculty member as least important in the group process of small-group learning [6], and several researchers have found that students prefer peer-led groups [6-10,20-22]. Student behavior and group functioning appears to be influenced by their perception of the learning situation [23], which suggests that students might do better in small group situations led by peers. Group functioning, in turn, is correlated with academic performance [19].

In these reports, key characteristics of the faculty-led or student-led groups (i.e., the students involved and the nature of the group members’ interactions) were not described. Adult Learning Theory describes the benefits of active learner involvement in all steps of learning [24], and as described in Self Directed Learning Theory, students are more engaged and active when they are responsible for their learning [25], leading to better learning outcomes [26]. Therefore, identifying teaching strategies consistent with these theories will promote learner engagement and outcomes. Our faculty made anecdotal observations that student discussion appeared to have increased in their absence when they stepped out of the room and then returned. Faculty noted that they often returned to more robust discussion than before they had left. Therefore, we decided to design a study to analyze student report and perceptions of group dynamics and group learning process within the same small group of students, comparing student report and perceptions of group dynamics and group learning process with the faculty member present and absent from the discussion. Examining these results will add to and strengthen what is known about the role of the faculty member in small group discussions, particularly how their presence or absence affects group learning and group dynamics.

## Methods

### Student population

All students rotating through the Boston University Family Medicine Clerkship between 7/21/06 and 5/15/07 were asked to participate in the study. Students were not required to participate, and clerkship faculty members were blinded as to which students completed surveys. Students who participated were told to label both their surveys with a random code that contained no information that might identify them. Students were read a standard informed consent informing them that they were being asked to participate in a survey related to group dynamics that might be used to improve instructional quality in the future. They were also clearly informed that participation was voluntary, anonymous and would not influence their grade.

During the academic year in which this study was conducted, there were 139 students who rotated through the Family Medicine clerkship, all of whom were invited to participate. Students were at various points in their third year, as this study was conducted over the course of a year, with students constantly rotating through this clerkship. The class was 46% male and 53% female; racial/ethnic data is unavailable, and survey participants were not asked to identify their gender, ethnicity or race on their survey. All students were enrolled at Boston University; no one was from out of town or participating in visiting clerkships.

### Setting and curriculum

Boston University School of Medicine requires a six week clerkship in Family Medicine, which all students rotate through at some point during their third year. During the Family Medicine clerkship, students return to the medical school for four days for didactics. The didactic curriculum is structured around two simulated families, with students managing and following multiple family members over time through the use of case studies related to various members of the fictional families. These case studies illustrate common out-patient issues routinely seen in family medicine, such as hypertension, family planning and diabetes. Case studies are explored during a series of small group sessions, in which students are randomly divided into three small groups by an administrative assistant, each with a faculty member. All small groups meet simultaneously. In the small groups, students are given paper cases for each family member’s doctor visits, which include history, physical exam, and laboratory data. Students work through the cases in a structured format: first they discuss the patient’s concerns and then they discuss the physician’s concerns. They then generate an assessment and plan for each visit. One student volunteers to scribe the group’s thoughts and plans on the blackboard. No other student roles are assigned. Faculty review the students’ assessment and plan, as written by the students on the blackboard, and direct the group to correct any mistakes.

During the course of the six week clerkship, each of the three small groups of students meet four times, with 4 to 5 cases discussed per session. Small group sessions last 3–4 hours. Student small groups are comprised of 6–8 students each, and the group composition remains constant. Cases are discussed in the same order in each group, as faculty members leading the groups are instructed to follow the order given in a standardized faculty guide.

There are four small group sessions during each clerkship block, with three small groups of students meeting simultaneously during each of those small group sessions. An administrative assistant randomly assigned which small group sessions in each block were to be studied. All small group sessions and all small groups of students were assigned to be studied at some point over the course of the study. In each of those small group sessions, students completed two surveys—one after a case when the faculty was in the room (“in”) and one after a case when the faculty had left the room for the bulk of the case discussion (“out”). Before leaving the room, the faculty gave the students scripted instructions, and left the room for a standardized amount of time. We rotated which cases were used as interventions (“out”) and controls (“in”).

We used the small group discussion structure that was explained to students prior to their first small group session and used in all small group case discussions, during cases that were studied. Students were told at the start of group work that they were being asked to participate in a voluntary, anonymous study that might be used to improve educational experiences in the future. This study design was quasi-experimental, as the same students participated and evaluated both formats of case discussions. Students were further informed that participation would not affect their grade. Students were not made aware that they were participating in a study of faculty “in” vs “out” of the room. Faculty members were given scripted language to use when they stepped out of the room explaining that they needed to step out for about 15 minutes (no reason for the absence was given) and instructing the students to continue with case discussion as before. Faculty left the room at the beginning of the case discussion. Each case is typically discussed for 20–40 minutes, varying based on the nature of the case and the speed of each particular small group of students.

Surveys were randomly distributed to each group at different times. An administrative assistant with no other role in the study was instructed to randomly include a survey packet in with the general instructional packets she distributed to each group at the start of each session. Groups would then discuss the cases already slated to be discussed that day, in the pre-determined order used by all groups. Each group was surveyed twice. While cases were not used in this study at the same time, over the course of the 8 block year, each case was discussed with faculty in and with faculty out at least once. (Certain cases include a role play where the faculty member plays the part of the patient. These cases require the presence of faculty and as such were excluded from the study. Students were not aware of the differentiation of these cases, and no groups were surveyed about these “faculty-required” cases). The difficulty level of all cases is felt to be equivalent.

### Instrument

**Figure 1:**
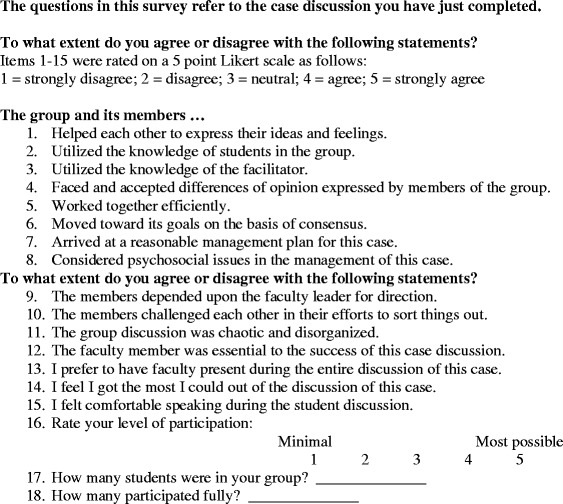
**Survey instrument.**

We used a survey instrument derived from the Group Climate Questionnaire – Short version (GCQ-S) [27], a validated instrument used previously by other researchers. Three items from the GCQ-S were used. In addition, eight items were used from Steele et al [17] in order to create an instrument more useful for our purpose [17]. Using the survey, participants self-scored and self-rated learning, group participation and dynamics using 16 Likert scaled questions. In addition, they were asked how many students were in their group and were asked to report their perceptions of how many students they felt participated fully. (See Figure 1). Students’ “in” surveys were then compared to their “out” surveys to assess differences in perceived group process and dynamics.

### Analysis

We performed a paired t-test analysis for each item to assess the change between the “in” and “out” score. SAS was used for all analyses (version 9.2, SAS Institute, Inc, Cary, NC). As there were 16 Likert-scaled items on the scale, we also performed a Bonferroni adjustment for multiple comparisons, dividing .05 by 16 and yielding a significance level of 0.0031. The Bonferroni adjustment is extremely conservative and led to some results being described as having a ‘trend’ toward significance (with p values less than .04 but more than .0031) [28].

This study was reviewed and approved by the Boston University research ethics Institutional Review Board (IRB).

## Results

**Figure 2:**
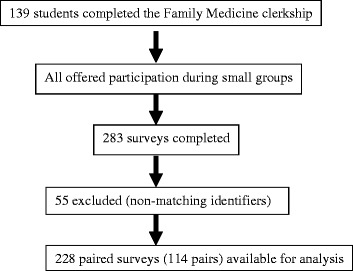
**Recruitment and enrollment.**

During the academic year in which this study was conducted, there were 139 students who rotated through the Family Medicine clerkship, all of whom were invited to participate. We collected 283 surveys in total. Of these, 228 had a pair, leading to 114 paired surveys, which were analyzed. In addition, we collected 55 surveys that did not have a pair, and so were excluded from analysis. We think this is the result of non-matching identifiers (i.e. student completed 2 surveys but failed to use the same identifier on each) as well as the possibility that some students chose to only complete one survey. (See Figure 2).

Students completed surveys at the completion of each case. They rated the entire case discussion – including time when the faculty was out of the room and time when the faculty was back in the room. Students self-rated the questions assessing and quantifying various elements of group dynamics/group learning process more highly during cases when faculty members were absent, stating that they felt the discussions were more participatory, used the knowledge of students within the group, and involved the members of the group challenging one another (Table 1). There was a trend towards rating the discussion as more “chaotic and disorganized” when faculty members were absent (p = 0.06). They were less likely to respond that they used the facilitator’s knowledge or relied upon the facilitator when the faculty member was absent, suggesting that faculty-absent groups might be more likely to motivate students towards the kind of critical thinking and responsibility needed by medical professionals.

**Table 1:** **Students’ perception of group dynamics/learning process, their participation, and case outcomes with faculty in and out of the discussion (n = 114)**

	**Mean score**		**p**
	**Faculty present “In” (n = 114)**	**Faculty absent “Out” (n = 114)**	
Student perception of group dynamics and group learning process			
*The group and its membez*			
Helped each other to express their ideas and feelings.^a^	4.4	4.6	0.015
Utilized the knowledge of students in the group.^a^	4.5	4.7	<0.01
The members challenged each other in their efforts to sort things out.^a^	4.1	4.4	0.012
Utilized the knowledge of the facilitator.^a^	4.2	3.5	<.001
The members depended upon the faculty leader for direction.^a^	3.3	2.3	<.001
The group discussion was chaotic and disorganized.^a^	1.4	1.6	0.06
Faced and accepted differences of opinion expressed by members of the group^a^	4.5	4.5	0.058
Worked together efficiently^a^	4.6	4.6	0.67
Moved toward its goals on the basis of consensus^a^	4.6	4.6	0.32
Student participation and preference			
Rate your participation level.^b^	4.2	4.3	0.03
I felt comfortable speaking during the student discussion.^a^	4.6	4.6	0.49
I prefer to have faculty present during the entire discussion of this case.^a^	3.5	3.1	0.0008
Case content			
Arrived at a reasonable management plan for this case.^a^	4.6	4.5	0.03
The faculty member was essential to the success of this case discussion.^a^	3.3	2.5	<.001
I feel I got the most I could out of the discussion of this case.^a^	4.3	4.1	0.67
Considered psychosocial issues in the management of this case.^a^	4.4	4.4	0.89

^a^Items are on a 5 point Likert scale as follows: 1 = strongly disagree; 2 = disagree; 3 = neutral; 4 = agree; 5 = strongly agree.

^b^Items are on a 5 point Likert scale as follows: 1 = Minimal; 5 = Most possible.

Regarding student participation, the students also rated their participation level and their comfort in participating more highly when the faculty member was absent. They were less likely to say that they preferred having the faculty member in the room for the entire case following an “out” case.

When we assessed case content (the outcome of the discussion), students were less likely to say that they arrived at a reasonable management plan after a case when the faculty was absent. However, when students rated whether they had gotten all they could out of the experience, there was no statistically significant difference between cases with faculty present or absent. Students were more likely to rate the faculty member’s presence as “essential” after a case with the faculty present. Finally, the students’ assessment of whether psychosocial issues were considered during the discussion was no different whether the faculty member was present or absent.

## Discussion

This study examined student report of group dynamics and group learning process with the faculty member present or absent from the discussion. We found that students have higher self-rated group dynamics, group learning process, and participation with the faculty member absent. Results on several domains (preference for faculty presence, arriving at a reasonable management plan for the case) suggest that students feel somewhat dependent on the faculty member, more so after a case when the faculty was present. However, after cases when the faculty was absent, students felt that the faculty member was *not* essential to the success of the case. And after cases when the faculty was present, students were only slightly in agreement that the faculty member was essential (3.3 on the same Likert scale). Additionally, there was no difference in how much they felt they got out of the case based on faculty presence or absence.

Given the goal of maximizing student learning, skill acquisition, and group experience, it is critical to understand the processes at play in the small group learning setting. The cognitive processes involved in small group learning situations can lead to better activation of prior knowledge, information recall, concept and theory building, and collaborative learning [29]. Likewise group discussion has been shown to motivate students and increase their interest in the subject matter [29] further enhancing student engagement and learning. Some studies suggest that higher social networking and peer interactions, both formal and informal, can increase learning [30] as well. Additionally, it has been shown that educational interventions regarding group dynamics and group function can improve small group effectiveness [31].

With the current emphasis on active learning, retention of material, and learner satisfaction associated with small group learning [3-5], the structure, process, and composition of the small group must also be better understood to promote these goals. Prior studies of the role of the faculty member in small group learning in medical education have shown mixed results with some showing better test scores, OSCE performance, and student satisfaction with an active faculty member [12-14], and others showing that a more active faculty member impedes the group learning process [16,18]. While many of the previous studies looked at faculty-led *or* student-led groups, this study compared self-reports from the same students in both faculty-led *and* student-led groups. Comparing self-reports from the same students in different kinds of groups showed that small groups without a faculty member present leads to better perceived group dynamics, group learning process, and increased student participation.

This study had several limitations. Students were recruited throughout the academic year, meaning some of the students in the study were near the end of their third year and more likely to be confident in their clinical knowledge and also had had more experience participating in small group discussions compared with early third-year students. The experiences that were compared using paired t-tests were group discussions within the same group of students and faculty member. However, the cases were different, which may have been partly responsible for some of the differences in scores (for example, a case featuring domestic violence might engender more or less animated discussion than a case featuring diabetes). In addition, faculty members leading groups had different amounts of experience and skill leading small group learning sessions. Because groups met simultaneously, we were not able to compare multiple groups with the same faculty member leading. However, evaluating groups with each faculty member in and out as well as conducting this study over the course of the year may help us address this limitation. Additionally, the cases used as intervention and control were rotated randomly. Finally, while many of the items in our survey had statistically significant differences showing increased discussion and participation when the faculty was absent, some of the absolute differences in scores were small.

This study implies that while we assume that students may have a tendency or desire to be more dependent on faculty, the critical activity of discussion and debate of clinical cases may necessitate structuring our curricula, methods, faculty development and/or educational design of our sessions to further promote and enable student participation and discussion. Next steps from this study include assessing learning outcomes such as test scores or clinical performance. Future study could build on these findings by obtaining student permission to record conversation in faculty-present and faculty-absent groups. This would enable a comparison of student participation and content using qualitative and quantitative methods.

## Conclusions

In this study, student small groups without faculty present reported better group dynamics, group learning processes, and participation with faculty absent. Our respondents reported that they feel somewhat dependent on faculty, especially when the faculty is present, though they report similar case success after both “in” and “out” cases. Further research into best practices and effective teaching methodology for small groups is called for in order to maximize student satisfaction and learning. As a result of these findings, we now encourage faculty to use stepping out of the room as a tool to increase small group discussion. This study has made us devote more faculty development time and discussion to this and other techniques to enhance small group participation. These results can guide changes to teaching modalities and methodologies as well as lead to redistribution of faculty time and resources in response to self-reported student needs. Clerkships may choose to have more small group sessions without faculty presence, or with intermittent faculty presence.
